# Engineering the plant holobiont for enhanced abiotic stress resilience: current insights and future directions

**DOI:** 10.1080/15592324.2026.2752710

**Published:** 2026-10-28

**Authors:** Malvin Albert, Mehrdad Alizadeh, Thomas Argyarich Jefferson, Dedat Prismantoro, Reginawanti Hindersah, Ravindra Chandra Joshi, Wiwiek Harsonowati, Kah-Ooi Chua, Nandang Permadi, Mia Miranti, Febri Doni

**Affiliations:** a Department of Biology, Faculty of Mathematics and Natural Sciences, Universitas Padjadjaran, West Java, Indonesia; b Department of Soil Science, University of Tehran, Tehran, Iran; c Doctorate Program in Biotechnology, Graduate School, Universitas Padjadjaran, West Java, Indonesia; d Department of Soil Science and Land Resources, Faculty of Agriculture, Universitas Padjadjaran, West Java, Indonesia; e Philippine Rice Research Institute, Maligaya, Science City of Muñoz, Nueva Ecija, Philippines; f Research Center for Horticulture, National Research and Innovation Agency (BRIN), West Java, Indonesia; g Institute of Biological Sciences, Faculty of Science, Universiti Malaya, Kuala Lumpur, Malaysia; h Centre for Research in Biotechnology for Agriculture (CEBAR), Universiti Malaya, Kuala Lumpur, Malaysia; i Research Center for Food Crops, Research Organization for Agriculture and Food, National Research and Innovation Agency (BRIN), West Java, Indonesia

**Keywords:** Synthetic microbial communities (SynComs), plant–microbe interactions, stress adaptation in plants, plant microbiomes, symbiotic lifestyle

## Abstract

Climate change has intensified abiotic stresses such as drought, heat, and salinity, posing major threats to agricultural productivity and global food security. Conventional strategies to improve crop tolerance often face limitations in efficiency, sustainability, and environmental adaptability, underscoring the need for innovative approaches. This review focuses on the plant holobiont, which views plants and their associated microbiomes as an integrated functional unit in governing stress adaptation. Abiotic stress can disrupt plant-associated microbial communities, leading to dysbiosis and reduced holobiont resilience. Beneficial microorganisms, including plant growth-promoting rhizobacteria, endophytes, and arbuscular mycorrhizal fungi, can enhance stress tolerance by improving nutrient acquisition, regulating phytohormone signaling, strengthening antioxidant defenses, maintaining osmotic balance, and modulating stress-responsive gene expression. Emerging evidence suggests that these interactions contribute to the establishment of holobiont stress adaptation through biochemical signaling, microenvironmental modification, and epigenetic regulation, enabling plants to respond more effectively to recurrent stress. Advances in microbiome engineering, particularly through the development of synthetic microbial communities (SynComs), offer promising opportunities to restore microbial functions and enhance crop resilience under changing climatic conditions. The integration of multi-omics technologies, artificial intelligence, and precision agriculture is expected to accelerate the development of microbiome-based solutions for sustainable crop production. Collectively, microbiome engineering represents a promising, environmentally sustainable strategy to enhance the holobiont’s adaptive resilience and improve long-term plant performance under abiotic stress.

## 1. Introduction

Climate change has become a major challenge for global agriculture, as it increases the occurrence and intensity of abiotic stresses such as drought, heat, salinity, flooding, and nutrient imbalance.^1^ ^,^ ^2^ These challenges threaten crop productivity, ecosystem stability, and food security, despite the need for a 50–60% increase in global food production by 2050.^1^ ^,^ ^3^ ^,^ ^4^ Abiotic stress occurs when adverse environmental conditions exceed the physiological tolerance of plants, disrupting cellular homeostasis and triggering complex physiological, biochemical, molecular, and genetic responses.^5‐8^ Despite their diverse origins, abiotic stresses commonly induce membrane damage, protein and nucleic acid degradation, lipid peroxidation, excessive reactive oxygen species (ROS) accumulation, and metabolic dysfunction, ultimately reducing plant growth and crop productivity.^9‐11^

Various strategies, including conventional breeding, molecular breeding, genetic engineering, agronomic management, and plant biostimulants, have been established to improve crop tolerance to abiotic stress.^12^ ^,^ ^13^ However, these approaches are often constrained by genotype dependency, environmental variability, high implementation costs, and sustainability concerns.^14^ Consequently, microbiome-based strategies have emerged as promising approaches for enhancing plant resilience.^15^ ^,^ ^16^ Plant-associated microorganisms inhabiting the rhizosphere, endosphere, and phyllosphere promote plant adaptation through nutrient acquisition, phytohormone regulation, osmolyte production, antioxidant defense, and stress-responsive gene regulation.^12^ ^,^ ^17^ These microorganisms establish complex associations with their host plants, collectively improving nutrient-use efficiency, growth, and stress tolerance.^17^ ^,^ ^18^ Nevertheless, the composition and functions of plant-associated microbial communities are highly dynamic and are frequently disrupted under abiotic stress, leading to alterations in microbial diversity and ecological functions.^15^ ^,^ ^19^ Conversely, host-derived signals can regulate microbial recruitment and community assembly, highlighting host signaling as an important component of plant–microbiome interactions. However, the mechanisms underlying host signaling and its role in shaping plant-associated microbial communities remain incompletely understood, particularly under changing environmental conditions.^20^

Plants and their associated microorganisms are increasingly recognized as a holobionts, an integrated ecological and evolutionary unit in which coordinated interactions between the host and microbiota determine plant fitness and environmental adaptation.^21‐24^ Within this system, plant-growth-promoting microorganisms (PGPMs) inhabiting the rhizosphere, phyllosphere, and endosphere enhance nutrient acquisition, stress tolerance, and disease resistance.^21^ ^,^ ^22^ ^,^ ^25^ ^,^ ^26^ Accordingly, plant resilience under abiotic stress depends on the dynamic interplay between host signaling, microbial recruitment, and the functional activities of associated microbial communities.^27^ ^,^ ^28^

However, despite rapid advances in plant microbiome research, the mechanisms linking host stress signaling, microbiome assembly, and microbiome engineering to plant resilience remain insufficiently synthesized. Although beneficial microorganisms, microbial inoculants, and synthetic microbial communities (SynComs) have been increasingly studied, their integration within the plant holobiont under abiotic stress remains poorly understood. The bibliometric analysis indicates that host signaling remains a relatively underexplored theme in plant–microbiome research, highlighting the need for further investigation of its role in microbiome assembly and plant resilience under abiotic stress. Therefore, this review synthesizes current knowledge of plant holobionts under abiotic stress, examines the links between host signaling and microbiome assembly, and evaluates advances in microbiome engineering, particularly SynComs, toward climate-resilient agriculture. This review hypothesizes that integrating host signaling and microbiome engineering can enhance plant holobiont resilience to abiotic stress through coordinated regulation of microbial functions and host stress responses.

## 2. Methodology of the review

### 2.1. Literature search and PRISMA screening

This review employed a structured literature search and screening process following the preferred reporting items for systematic reviews and meta-analyzes (PRISMA) framework. Relevant literature was identified from Scopus, Web of Science, PubMed, Semantic Scholar, and Google Scholar using combinations of keywords related to “plant microbiome”, “plant–microbe interaction”, “microbiome engineering”, “abiotic stress”, “stress resilience”, “root microbiome”, and “microbial community”. A total of 500 records were initially identified. After removing 35 duplicates, 465 records were screened based on their titles and abstracts. Studies unrelated to plant microbiomes, abiotic stress, plant–microbe interactions, or microbiome engineering were excluded. A total of 227 articles assessed for eligibility, 80 were excluded because they were not in English, were not peer-reviewed, or had unavailable full texts. Finally, 147 studies were included in the qualitative synthesis. The complete study selection process is presented in Figure 1.

**Figure 1.**
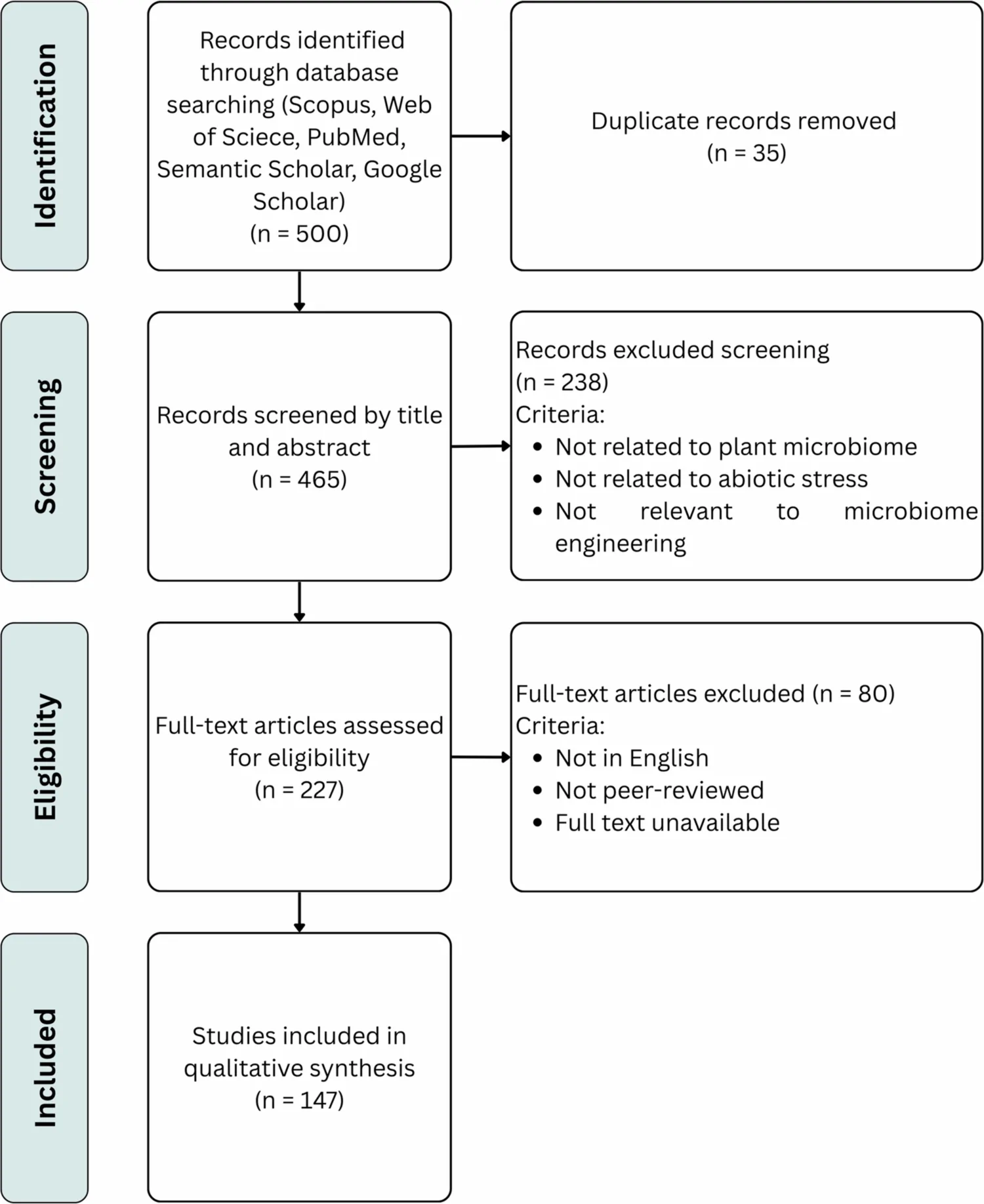
PRISMA flow diagram of the literature search and selection process for studies on plant microbiome and stress resilience.

### 2.2. Bibliometric analysis and thematic synthesis

Bibliometric analysis was performed using VOSviewer version 1.6.20 to identify major research themes and conceptual relationships within the selected literature. Keyword co-occurrence analysis was used to visualize the structure of the research field. The resulting network comprised 38 terms, 403 links, and a total link strength of 1,805, grouped into five thematic clusters (Figure 2). The network highlighted prominent connections among stress resilience, plant microbiome engineering, plant–microbe interaction, abiotic stress, root, soil, nutrient cycling, microbial communities, and plant health. These patterns were used to guide the thematic synthesis and identify research gaps. Notably, the bibliometric landscape suggests a comparatively weak integration between microbiome engineering/stress resilience and the signaling processes linking stress perception, host molecular responses, chemical-mediated microbial recruitment, community assembly, microbial feedback, and epigenetic priming. This gap was therefore used to frame the subsequent synthesis of mechanisms underlying plant–microbiome-mediated stress resilience.

**Figure 2.**
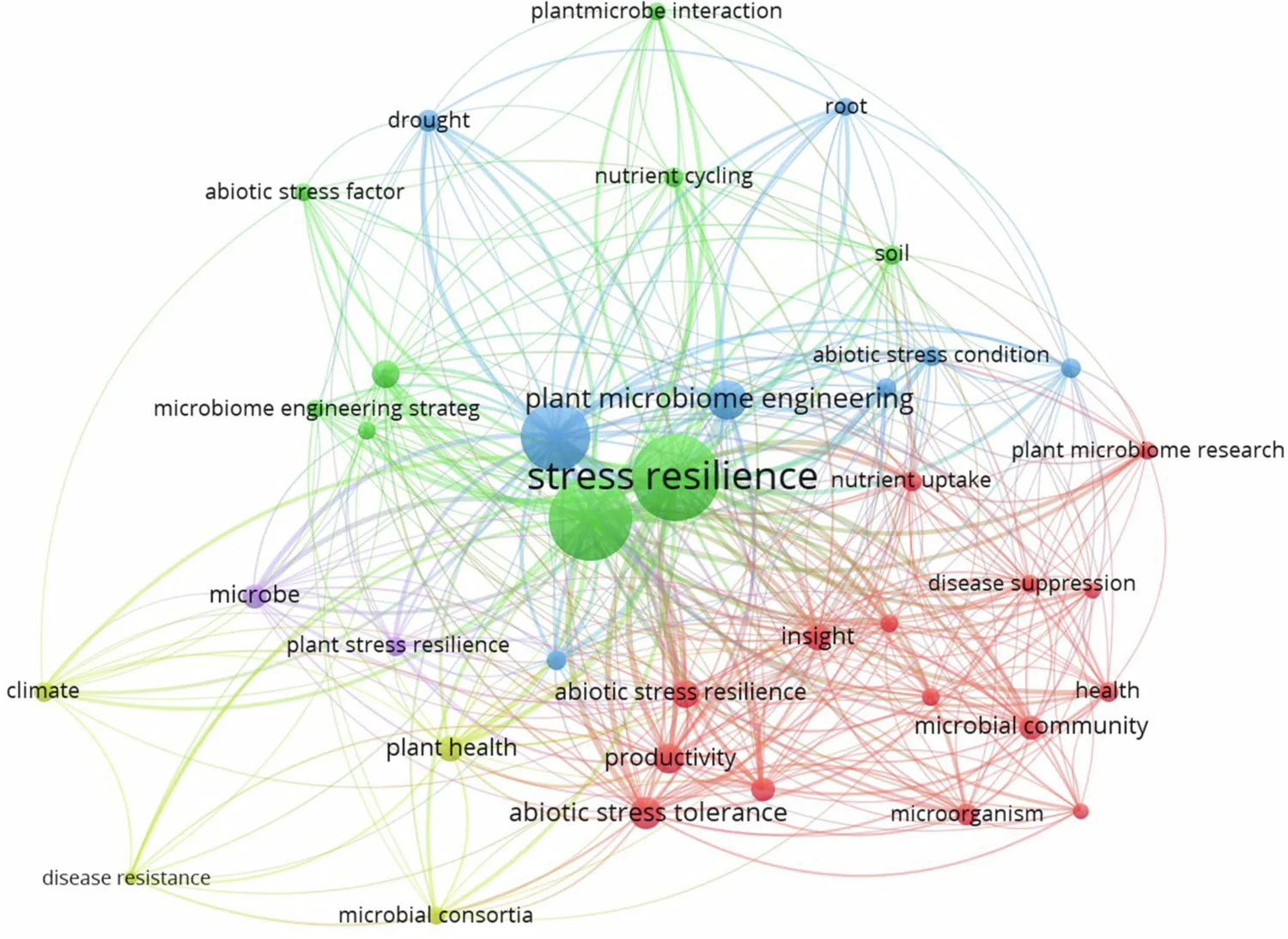
Bibliometric co-occurrence network illustrating the major research themes and conceptual relationships within plant microbiome engineering and stress resilience.

## 3. Plant holobiont: concept and functional significance

The concept of the plant holobiont has fundamentally changed how plants are perceived, shifting from independent organisms to integrated biological systems composed of the host plant and its associated microbiota.^21^ ^,^ ^29^ The holobiont concept describes the host and its associated symbiotic microorganisms as an ecological and evolutionary unit, whereas the hologenome refers to the collective genetic information of both partners.^30^ ^,^ ^31^ In plants, these microbial communities inhabit the rhizosphere, endosphere, and phyllosphere, where they establish dynamic interactions that influence plant development, nutrient acquisition, metabolism, and responses to environmental change.^26^ ^,^ ^32^ Rather than acting independently, plants and their associated microorganisms operate as a coordinated system in which microbial activities complement host physiological processes.^26^ ^,^ ^30^ ^,^ ^33^ Consequently, plant phenotype, performance, and adaptation should be considered as emergent properties of the entire holobiont that involves interactions between the plant genome and its hologenome, rather than the host genome alone.^30^ ^,^ ^31^ However, the holobiont concept should be applied carefully because the stability, heritability, and functional integration of host-associated microbiomes may vary across plant species, developmental stages, soil conditions, and environmental contexts.^34^ ^,^ ^35^ Thus, rather than treating the plant holobiont as a fixed evolutionary unit, it may be more appropriate to view it as a dynamic ecological system whose adaptive functions depend on context-specific plant–microbe interactions and the capacity of microbial communities to maintain functional stability under environmental stress.^36^ ^,^ ^37^

Under abiotic stress conditions, the functional significance of the plant holobiont becomes increasingly evident.^38^ ^,^ ^39^ Environmental stresses not only affect plant physiology but also reshape the diversity, composition, and metabolic activity of plant-associated microbial communities.^39^ ^,^ ^40^ In turn, beneficial microorganisms contribute to plant adaptation by improving nutrient availability, regulating phytohormone balance, maintaining redox homeostasis, enhancing osmotic adjustment, and modulating stress-responsive signaling pathways.^39^ ^,^ ^41^ These reciprocal interactions enable the holobiont to maintain physiological stability and improve plant resilience under adverse environmental conditions.^38^ ^,^ ^41^ However, because these interactions are highly dynamic, environmental disturbances that alter microbial community structure may impair holobiont functionality, reducing the plants’ capacity to tolerate prolonged or repeated abiotic stress.^27^ ^,^ ^42^

Recognizing plants as holobionts provides an important conceptual framework for developing microbiome-based strategies to improve crop resilience.^29^ ^,^ ^43^ ^,^ ^44^ Instead of focusing solely on the host plant or individual beneficial microorganisms, this perspective emphasizes the collective functions and interactions of the entire plant-associated microbial community.^29^ ^,^ ^43^ ^,^ ^45^ The integrated perspective has stimulated growing interest in microbiome engineering, including the design of SynComs, to restore or optimize holobiont functions under stressful environments.^43^ ^,^ ^46^ ^,^ ^47^ Therefore, understanding the structure, function, and stability of the plant holobiont is essential for explaining how abiotic stress disrupts plant–microbiome interactions and for developing sustainable strategies to enhance crop adaptation under changing climatic conditions.

## 4. Dysbiosis in plant microbiomes

Plant-associated microbial communities normally exist in a dynamic but relatively stable equilibrium, where interactions among microorganisms and their host collectively support nutrient acquisition, growth, disease suppression, and environmental adaptation.^15^ ^,^ ^26^ ^,^ ^28^ This balanced state is essential for maintaining the functional integrity of the plant holobiont.^24^ ^,^ ^48^ ^,^ ^49^ However, disturbances that alter microbial composition or function can disrupt these interactions, resulting in a condition known as dysbiosis.^48^ ^,^ ^50^ In plants, dysbiosis refers not only to changes in microbial diversity and abundance but also to the loss of beneficial microbial functions, disruption of ecological interactions, and reduced stability of the microbial community.^48^ ^,^ ^50^ Unlike healthy microbiomes, dysbiotic communities are characterized by reduced functional redundancy. This parameter is quantitatively evaluated using the ecological alpha and beta diversity indices of predicted metabolic pathways derived from high-throughput metagenomic datasets, such as the Shannon index applied to metabolic functional groups, or measured through community-level physiological profiling (CLPP) indices that assess substrate utilization patterns over time. Furthermore, functional redundancy can be calculated computationally by mapping taxonomic abundance against metagenomic metabolic flux models to quantify the number of distinct taxa contributing to the same ecological function, such as nitrogen fixation or phosphorus solubilization. These communities are further impacted by fragmented microbial network connectivity and an increased susceptibility to the dominance of opportunistic pathogens, which ultimately compromises host performance.^51^

From a community ecology perspective, abiotic stresses represent a primary catalyst for dysbiosis by altering the underlying niche space within the plant structures.^52‐54^ When extreme environmental pressures cross critical tipping points, the collective community experiences an ecological regime shift that compromises network connectivity.^55^ ^,^ ^56^ These structural disturbances frequently compress taxonomic diversity, deplete critical keystone taxa, weaken competitive ecological interactions, and shift the balance of microbial metabolic investments away from mutualistic symbiosis toward individual cellular survival.^51^ ^,^ ^57^ Moreover, prolonged or recurrent stress may reduce the resilience of microbial communities, making it increasingly difficult for the plant holobiont to recover its original functional state following environmental disturbance.^15^ ^,^ ^48^ ^,^ ^58^ Hence, abiotic stress should be regarded not only as a direct constraint on plant physiology but also as a major ecological force that reshapes plant-associated microbiomes.^15^ ^,^ ^43^ ^,^ ^48^

The consequences of dysbiosis extend beyond taxonomic changes in microbial communities and directly influence the functional capacity of the plant holobiont.^48^ ^,^ ^51^ Disruption of beneficial microbial populations may reduce biological nitrogen fixation, phosphorus solubilization, phytohormone production, antioxidant activity, osmotic regulation, and natural suppression of soil-borne pathogens, thereby weakening plant growth and stress tolerance.^57^ ^,^ ^59^ ^,^ ^60^ At the ecosystem level, dysbiosis can also impair nutrient cycling, soil fertility, and microbial interactions that support ecosystem stability.^51^ Because the adaptive capacity of plants depends on coordinated interactions between the host and its associated microbiota, dysbiosis should be viewed as a disruption of holobiont functionality rather than merely a shift in microbial community composition.^24^ ^,^ ^48^

More importantly, not all stress-induced changes in the plant microbiome should be considered dysbiosis. Some microbial changes may represent adaptive microbiome reassembly, such as the enrichment of stress-tolerant taxa or beneficial functional guilds under drought, salinity, or nutrient limitation.^52^ ^,^ ^54^ ^,^ ^61^ Therefore, dysbiosis should be distinguished from adaptive restructuring by considering whether the shift leads to the loss of beneficial microbial functions, reduced functional redundancy, weakened microbial networks, or increased dominance of opportunistic microorganisms.^56^ ^,^ ^62^ In agricultural systems, the loss of microbial functions related to nutrient acquisition, plant growth promotion, and natural suppression of soil-borne pathogens may weaken both crop productivity and holobiont resilience, highlighting the importance of maintaining beneficial microbial communities through sustainable microbiome-based strategies.^63^ ^,^ ^64^

## 5. Plant responses to abiotic stress

Abiotic stresses represent one of the primary environmental factors shaping plant growth, productivity, and microbiome assembly.^19^ Among these, elevated temperature, drought, and salinity are the most prevalent climate-driven stressors affecting agricultural systems worldwide.^65^ ^,^ ^66^ Because the root system represents the primary interface between the holobiont and edaphic stress factors, individual abiotic pressures trigger distinct, highly specific modifications in plant underground morphology and signaling. These stressors collectively disrupt plant–microbiome interactions by forcing structural alterations in host root anatomy, altering the qualitative and quantitative chemical profiles of root exudates, modifying soil physicochemical properties, and inducing a deterministic restructuring of the rhizosphere and endosphere niches.^64^ ^,^ ^67^ ^,^ ^68^ These environmental variations frequently lead to dysbiosis, which impairs the functional stability of the plant holobiont and reduces its overall capacity to cope with subsequent or concurrent environmental challenges.^64^ ^,^ ^68^ Elucidating how individual abiotic stressors influence plant-associated microbiomes is therefore essential for elucidating the mechanisms underlying plant adaptation and for developing effective microbiome-based strategies to improve crop resilience under changing climatic conditions.

### 5.1. Heat

Temperature plays an important role in regulating plant growth, development, and the composition of microbial communities associated with plants.^69^ Projected global warming is expected to intensify heat stress and alter the composition, diversity, and functional activity of rhizosphere microbial communities, with significant consequences for plant productivity and ecosystem stability.^19^ At the cellular level, elevated temperature disrupts membrane and protein homeostasis, triggering Ca^2+^ influx and ROS accumulation. These signals activate calcium-dependent protein kinases (CDPKs) and mitogen-activated protein kinase (MAPK) cascades, which regulate downstream heat-responsive transcription factors and stress-related genes.^70^ It also influences microbial carbon utilization and may increase the virulence of certain phytopathogens, thereby exacerbating disease severity under heat stress.^19^ Nevertheless, many microorganisms exhibit physiological acclimation that enables them to maintain metabolic activity under heat stress and fluctuating thermal conditions.^19^ ^,^ ^71^

Thermotolerant microorganisms have evolved diverse physiological and molecular adaptations that enable to survive at elevated temperatures while maintaining beneficial interactions with host plants.^72^ ^,^ ^73^ Thermotolerant microbes, including endophytic bacteria and arbuscular mycorrhizal fungi (AMF) can improve plant thermotolerance by maintaining nutrient uptake, modulating Ca^2+^ and ROS signaling, and enhancing antioxidant capacity under heat stress.^69^ ^,^ ^73^ ^,^ ^74^ The agricultural potential of thermotolerant microorganisms has been demonstrated across multiple crop species, where plant growth-promoting bacteria improve biomass accumulation, chlorophyll content, photosynthetic efficiency, and crop productivity under heat stress.^72^ ^,^ ^75^ ^,^ ^76^ Microbial signals can reinforce the HSFA1–HSFA2/HSFA3–HSE cascade, inducing heat-shock proteins such as HSP70, HSP90, HSP101, and small HSPs that stabilize proteins and maintain proteostasis under heat stress.^77^ Some beneficial microorganisms, such as *Burkholderia phytofirmans* PsJN, also confer cross-protection against multiple abiotic stresses, highlighting their potential for climate-resilient agriculture.^19^ ^,^ ^78^

At the molecular level, beneficial rhizobacteria enhance plant thermotolerance by regulating interconnected Ca^2^⁺–ROS, antioxidant, and phytohormone signaling networks rather than acting through a single pathway.^79^ ^,^ ^80^ Heat-induced superoxide (O₂^-^) is converted to H₂O₂ by superoxide dismutase (SOD), while catalase (CAT) and ascorbate peroxidase (APX) detoxify H₂O₂ and limit oxidative damage. Beneficial microorganisms can enhance these antioxidant systems while maintaining ROS levels required for signaling.^81^ Microbial modulation of abscisic acid (ABA), ethylene, and auxin signaling further contributes to thermotolerance by regulating stress-responsive gene expression, water balance, and root development.^81^ In particular, microbial indole-3-acetic acid (IAA) can promote root development, whereas 1-aminocyclopropane-1-carboxylate (ACC) deaminase-producing bacteria can reduce excessive ethylene accumulation by degrading its precursor ACC.^82^ Investigating plant–microbiome interactions helps identifying beneficial microbial taxa and developing microbiome-based strategies to improve crop resilience under future climate scenarios.^19^

These heat-induced microbial and transcriptional changes may contribute to thermal priming within the plant holobiont, allowing the host plant and its associated microorganisms to respond more rapidly to repeated heat exposure.^83‐85^ Such priming may involve the persistence of thermotolerant microbial taxa, enhanced antioxidant capacity, altered hormone responsiveness, and faster activation of the Ca^2^⁺–ROS and HSF–HSP pathways during subsequent heat exposure, which collectively support the holobiont adaptation under recurrent heat stress.^86^ ^,^ ^87^

### 5.2. Drought

Drought represents one of the most critical abiotic stresses restricting global agricultural productivity, with projections indicating it will become more frequent and severe due to climate change.^88^ ^,^ ^89^ Water deficit disrupts plant water status, causing stomatal closure, reduced photosynthetic activity, impaired nutrient uptake, inhibited cell expansion, and decreased biomass accumulation.^90^ At the cellular level, drought induces osmotic stress, excessive accumulation of ROS, membrane lipid peroxidation, protein oxidation, and metabolic dysfunction, ultimately reducing crop yield and quality.^91^ ^,^ ^92^ To survive these conditions, plants activate complex adaptive responses, including osmotic adjustment through compatible solute accumulation, the upregulation of antioxidant defenses, phytohormone-mediated signaling, and root system modification to enhance water acquisition.^93^ ^,^ ^94^ A central drought-response pathway is mediated by ABA, which activates the PYR/PYL–PP2C–SnRK2 cascade and downstream ABF/AREB transcription factors, inducing genes involved in stomatal regulation, osmotic adjustment, and stress protection.^82^ ^,^ ^95^

Beyond its direct effects on plant physiology, drought profoundly alters the structure and function of plant-associated microbial communities.^96^ ^,^ ^97^ Reduced soil moisture, changes in soil physicochemical properties, and drought-induced alterations in root exudation modify microbial recruitment and community assembly, frequently reducing microbial diversity while enriching drought-adapted taxa such as Actinobacteria and Firmicutes.^98‐100^ These shifts influence microbial metabolic activity, ecological interactions, and nutrient cycling, potentially disrupting beneficial plant–microbe associations but also promoting microorganisms capable of enhancing plant adaptation under water-limited conditions.^101‐105^

Beneficial microorganisms alleviate drought stress through multiple complementary mechanisms that collectively improve plant performance.^106^ ^,^ ^107^ Plant growth-promoting rhizobacteria (PGPR), Actinobacteria, and AMF enhance nutrient acquisition, stimulate root growth, regulate phytohormone balance, produce osmoprotectants and exopolysaccharides, and express ACC deaminase, thereby improving water-use efficiency and reducing drought-induced oxidative damage.^105,106,108‐110^ Microbial IAA can promote root development and water acquisition, whereas ACC deaminase lowers excessive ethylene accumulation by degrading its precursor ACC, thereby reducing ethylene-mediated inhibition of root growth.^82^ Microbial exopolysaccharides can additionally improve soil aggregation and water retention around roots.^111^ These microorganisms also enhance SOD, CAT, and APX activities to control drought-induced ROS accumulation, thereby maintaining cellular redox homeostasis and reducing oxidative damage.^105^ ^,^ ^112^

Several microbial taxa have been consistently reported to enhance drought tolerance across diverse crop species.^113^ ^,^ ^114^ *Pseudomonas* spp. improve plant growth through ACC deaminase activity, phytohormone production, and exopolysaccharide synthesis, thereby promoting root development and maintaining water status under drought conditions.^115^ ^,^ ^116^ On the another hand, *Streptomyces* spp. contribute to drought adaptation by producing bioactive metabolites that stimulate root growth and antioxidant defenses.^117^ ^,^ ^118^ AMF further enhance drought tolerance by expanding the effective root absorptive surface through formation of extensive hyphal networks around the plant root regions, thus improving water and nutrient uptake, and strengthening plant physiological resilience under prolonged water limitation.^117^ ^,^ ^119^ ^,^ ^120^

Recurrent drought may generate microbial legacy effects in the rhizosphere, in which drought-adapted microbial taxa and functions persist after initial stress exposure and support improved plant performance during subsequent water limitation.^61^ ^,^ ^121^ ^,^ ^122^ Such microbial legacies may reinforce root development, osmotic adjustment, antioxidant capacity, and nutrient acquisition, potentially facilitating faster plant responses to subsequent drought exposure.^123‐125^

### 5.3. Salinity

Salinity is one of the major abiotic stresses that impacts global agricultural productivity worldwide, with increasing soil salinization accelerated by inappropriate irrigation management, climate change, and saline water intrusion^126‐128^ Excessive accumulation of soluble salts, particularly sodium (Na⁺) and chloride (Cl^−^), disrupts ionic homeostasis and water uptake, resulting in osmotic stress, ion toxicity, nutrient imbalance, and oxidative damage that collectively impair seed germination, photosynthesis, plant growth, and crop yield.^127,129‐131^ To withstand saline environments, plants activate multiple adaptive mechanisms, including osmotic adjustment through compatible solute accumulation, ion exclusion and compartmentalization, antioxidant defense systems, and phytohormone-mediated stress signaling.^132^ ^,^ ^133^ A key component of salt tolerance is Na⁺ homeostasis mediated by the salt overly sensitive (SOS) pathway, in which salt-induced Ca^2+^ signaling activates SOS3/SOS2, which in turn regulates the Na⁺/H⁺ antiporter SOS1 to promote Na⁺ extrusion. High-affinity K⁺ transporters (HKT) and Na⁺/H⁺ exchangers (NHX) further contribute to Na⁺ exclusion and vacuolar sequestration, respectively.^134^

Salinity also profoundly influences the structure and function of plant-associated microbial communities by altering soil physicochemical properties, microbial metabolism, and nutrient supply.^135^ High salt concentrations reduce microbial diversity and modify community composition, while simultaneously selecting for salt-tolerant microorganisms capable of surviving under osmotic and ionic stress^136^ ^,^ ^137^ These microbial shifts affect nutrient cycling, root colonization, and plant–microbe interactions, thereby influencing the functional stability of the plant holobiont under saline conditions.^138^ ^,^ ^139^

Beneficial microorganisms enhance plant salinity tolerance through complementary mechanisms that support ion homeostasis, osmotic adjustment, and redox balance.^140^ ^,^ ^141^ PGPR, endophytic bacteria, and AMF improve nutrient acquisition, regulate phytohormone balance, maintain ion homeostasis, stimulate antioxidant enzyme activity, produce osmoprotectants and exopolysaccharides, and express ACC deaminase, collectively reducing salt-induced oxidative damage and improving plant growth under saline conditions.^107^ ^,^ ^140^ ^,^ ^142^ Microbial inoculation can also strengthen Na⁺/K⁺ homeostasis by promoting ion exclusion or compartmentalization, while enhancing SOD, CAT, and APX activities to control salt-induced ROS accumulation.^143^ ^,^ ^144^ These responses collectively support photosynthetic performance and cellular stability under high salinity.

Several microbial taxa have consistently demonstrated the ability to improve plant performance under salt stress. Species of *Bacillus*, *Pseudomonas*, *Enterobacter*, *Sphingomonas*, and *Pantoea* enhance salinity tolerance by regulating phytohormone production, improving nutrient availability, and strengthening antioxidant defenses.^145‐147^ Likewise, *Serratia marcescens* VIRS2 has been reported to enhance salt tolerance in rice by increasing the accumulation of compatible solutes, including proline and other amino acids, through abscisic acid-mediated regulation.^148^ ^,^ ^149^ AMF further contribute to salinity tolerance by improving nutrient and water uptake, maintaining ionic balance, and enhancing plant physiological performance under saline environments.^150^

Such salt-induced microbial selection may contribute to holobiont adaptation by maintaining microbial functions associated with ion homeostasis, osmotic adjustment, antioxidant regulation, and phytohormone-mediated stress signaling across repeated saline conditions.^151‐153^ The persistence of salt-tolerant microbial taxa, including *Bacillus, Pseudomonas, Enterobacter, Sphingomonas, Pantoea, Serratia,* and AMF, may provide functional continuity that enables the plant holobiont to respond more efficiently to recurrent salinity stress.^154^ ^,^ ^155^

### 5.4. Combined and sequential abiotic stress: trade-offs in holobiont resilience

In natural environments, drought, heat, and salinity often occur simultaneously or sequentially, producing responses that differ from those induced by individual stresses.^156^ Under single stress, plants activate relatively specific responses: drought increases ABA-mediated stomatal closure to conserve water, heat promotes transpiration, and heat-shock protein (HSP) production, while salinity induces osmotic adjustment and Na⁺/K⁺ homeostasis.^77^ ^,^ ^95^ ^,^ ^144^ These stresses also increase ROS, which are detoxified through antioxidant systems such as superoxide dismutase (2O₂^−^ + 2H⁺ → H₂O₂ + O₂) and catalase (2H₂O₂ → 2H₂O + O₂).^81^ Under combined stress, however, these responses interact and may conflict, for example stomatal closure conserves water during drought but restricts evaporative cooling during heat, while drought–salinity requires simultaneous regulation of water status and ion toxicity.^157^ Thus, multiple-stress tolerance depends on coordinating overlapping and competing physiological responses rather than simply combining single-stress responses.

Stress signaling also reshapes plant–microbiome interactions.^158^ Changes in hormonal, redox, and metabolic signaling modify root exudates, including sugars, amino acids, organic acids, and flavonoids, which influence microbial recruitment and community assembly.^159^ Beneficial microorganisms can subsequently provide complementary functions through EPS production for rhizosphere water retention, IAA-mediated root development, nutrient solubilization, and hormone modulation.^160^ ^,^ ^161^ Microbial ACC deaminase can reduce stress-induced ethylene by converting ACC into *α*-ketobutyrate and ammonia (ACC + H₂O → *α*-ketobutyrate + NH₃), while microbial metabolites and VOCs can prime antioxidant and osmoprotective responses.^162^ Under combined stress, these functions may operate simultaneously to maintain water acquisition, nutrient and ion balance, and ROS homeostasis, indicating that microbial recruitment and function are shaped by the integrated stress state of the host rather than by individual stressors alone.

Sequential stresses add further complexity because the first stress can modify hormone signaling, metabolism, root exudation, and microbiome composition, thereby influencing the response to a subsequent stress.^163^ Such physiological and microbial priming can enable faster or stronger responses to the second stress.^164^ Consequently, holobiont resilience under multiple stresses emerges from dynamic interactions among plant signaling, microbial recruitment, functional complementarity, and physiological trade-offs. This suggests that microbiome engineering should prioritize microbial consortia with complementary functions that remain effective across interacting and temporally variable stresses rather than selecting microorganisms solely for single-stress tolerance.

Collectively, these studies demonstrate that although heat, drought, and salinity affect plants through distinct physiological and ecological pathways, beneficial microorganisms employ several conserved adaptive mechanisms, including phytohormone regulation, antioxidant activation, osmotic adjustment, and improved nutrient acquisition, to enhance plant resilience. A comparative summary of the microbiome-mediated mechanisms involved in plant adaptation to major abiotic stresses is presented in Table 1.

**Table 1.** Microbiome-mediated mechanisms involved in plant adaptation and potential holobiont resilience under major abiotic stresses.

Abiotic stresses	Microorganisms	Major mechanisms	Plant responses	References
Heat	*Burkholderia phytofirmans,* thermotolerant PGPR, AMF	Heat-shock protein induction, antioxidant activation, phytohormone regulation, improved nutrient acquisition	Enhanced thermotolerance, maintenance of photosynthesis, increased biomass and productivity	El-Tarabily et al. [74]; Macabuhay et al. [165]; Jaros-Tsoj et al. [166]; Chopra et al. [167]
Drought	*Pseudomonas* spp*., Streptomyces* spp., AMF	ACC deaminase activity, extracellular polymeric substances (EPS) production, osmoprotectant synthesis, antioxidant defense, phytohormone modulation	Improved water-use efficiency, enhanced root growth, osmotic adjustment, reduced oxidative damage	Ndlazi et al. [115]; Sharma et al. [168]; Etesami et al. [169]; Ghorui et al. [170]
Salinity	*Bacillus, Pseudomonas, Enterobacter, Sphingomonas, Pantoea, Serratia marcescens,* AMF	Ion homeostasis, abscisic acid (ABA) regulation, ACC deaminase activity, osmoprotectant production, antioxidant defense	Improved Na⁺/K⁺ balance, enhanced nutrient uptake, increased photosynthesis, improved salt tolerance	Zhang et al. [171]; Gao et al. [172]; Afzal et al. [173]

## 6. The plant microbiome as a key mediator of climate change adaptation

As climate change intensifies, plants face escalating abiotic stresses such as drought, heat, and salinity.^174^ ^,^ ^175^ Increasingly, research highlights the critical role of plant-associated microbial communities, the microbiome, in mediating plant resilience and adaptation to these challenges.^176^ ^,^ ^177^ Plants actively recruit beneficial microorganisms through dynamic changes in root exudation and molecular signaling pathways, a phenomenon known as the “cry for help” response.^177^ ^,^ ^178^

Plants actively recruit and shape their microbiomes by altering root exudates, hormone signaling, small RNAs, and epigenetic regulation, thereby enriching beneficial microorganisms that enhance stress adaptation.^179^ ^,^ ^180^ Plant-microbe interactions are established through diverse ecological relationships that collectively contribute to plant adaptation under environmental stress.^181^ ^,^ ^182^ Beneficial microorganisms, including AMF, rhizobia, PGPR, and endophytes, enhance plant fitness by improving nutrient acquisition, regulating metabolism, and strengthening stress tolerance.^109^ ^,^ ^183^ These microbiome-mediated interactions constitute an integrated adaptive framework through which beneficial microorganisms enhance plant resilience via engineering the plant microenvironment, biochemical signaling and hormonal modulation, and epigenetic regulation of gene expression, ultimately contributing to the establishment of plant holobiont resilient under abiotic stress (Figure 3).

**Figure 3.**
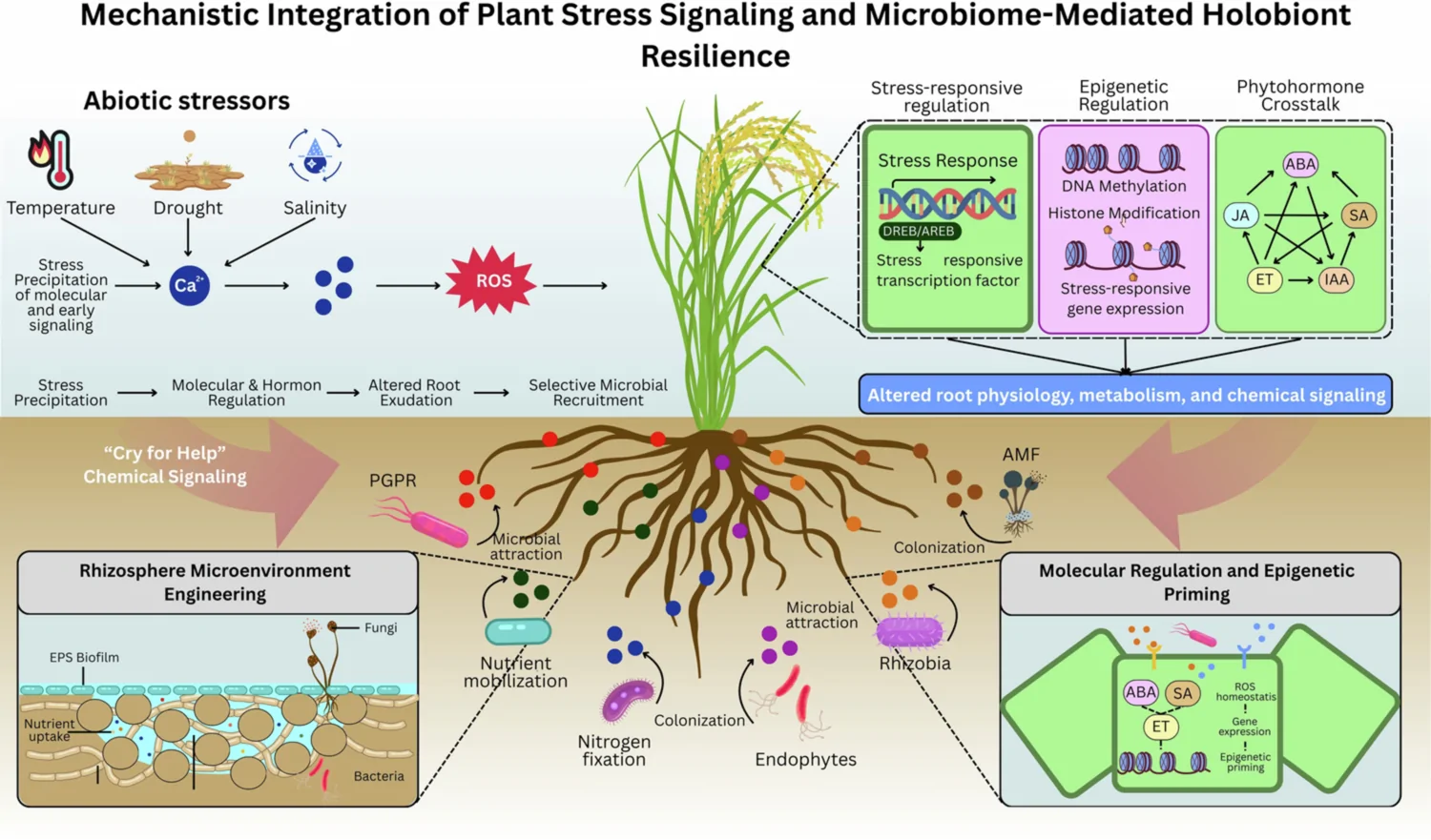
Conceptual framework microbiome-mediated mechanisms underlying plant adaptation and holobiont resilience under abiotic stress.

### 6.1. Engineering the plant microenvironment

Plant microbiomes actively modify and shape the physicochemical conditions of local environments surrounding the roots and leaves, thereby enhancing plant adaptation to environmental stress.^184^ Many beneficial bacteria produce EPS that facilitate biofilm formation, improve soil aggregation, increase water retention, and enhance root colonization.^185^ ^,^ ^186^ This effect can extend beyond physical water retention, as biofilm formation by *B. velezensis* D103 was associated with increased expression of the plant aquaporins ZmPIP2;6 and ZmTIP1;1, enhancing root water transport under drought.^187^ Similarly, AMF improve soil structure through glomalin production, whereas phyllosphere microorganisms influence leaf wettability and water balance, collectively enhancing plant adaptation to abiotic stress.^120^

These microbiome-mediated modifications create a more favorable rhizosphere and phyllosphere that supports plant physiological performance tolerance to environmental stresses.^188^ Under drought, EPS-mediated biofilms and AMF hyphae can maintain water availability around roots, whereas under salinity, microbial polymers may additionally bind or immobilize Na⁺ and modify its movement within the rhizosphere, reducing ionic exposure to roots.^189^ Improved soil structure enhances water infiltration, aeration, nutrient diffusion, and root proliferation while promoting the persistence and activity of beneficial microorganisms, thereby reinforcing positive plant–microbe interactions.^190^ ^,^ ^191^ Likewise, microbial biofilms and surface-associated metabolites on leaf surfaces can regulate foliar microclimatic conditions, reduce transpirational water loss, and improve leaf hydration during heat and drought stress.^192^ By collectively modifying the plant microenvironment, these microbial communities help maintain resource availability and physiological stability, ultimately strengthening the resilience of the plant holobiont under adverse environmental conditions.

### 6.2. Biochemical signaling and hormonal modulation

Microorganisms influence plant and enhances plant tolerance to abiotic stresses by producing phytohormones and signaling molecules or by regulating their biosynthesis within the host plant.^193^ ^,^ ^194^ These biochemical interactions stimulate plant growth, nutrient uptake, and stress adaptation through the production of auxins, cytokinins, gibberellins, siderophores, ACC deaminase, and volatile organic compounds (VOCs).^195^ ^,^ ^196^ For example, microbial IAA promotes lateral root formation and root elongation, increasing the capacity for water and nutrient acquisition, whereas ACC deaminase reduces excessive ethylene accumulation by degrading its precursor ACC, thereby preventing ethylene-mediated inhibition of root growth under stress.^197^ Furthermore, microbial metabolites such as 2,3-butanediol and coumarin-mediated signaling strengthen reciprocal plant–microbe communication and enhance tolerance to drought, salinity, and heat stress by activating multiple defense pathways.^198‐200^

Importantly, microbial hormonal effects interact with stress-specific plant signaling pathways rather than acting through a single universal mechanism. Under drought, microbial modulation of ABA homeostasis can influence the PYR/PYL–PP2C–SnRK2 pathway, which controls stomatal regulation and downstream stress-responsive gene expression.^95^ Under salinity, microbial-mediated changes in ABA signaling can act together with mechanisms controlling Na⁺/K⁺ homeostasis, whereas under heat stress, microbial signals can interact with ROS-, Ca^2+^-, ethylene-, jasmonic acid (JA)-, and salicylic acid (SA)-dependent responses to support cellular protection.^77^ ^,^ ^134^ Thus, microbial effects on phytohormones are integrated with, rather than independent of, the core stress-signaling networks.

Beyond individual signaling molecules, biochemical communication between plants and microorganisms involves highly coordinated molecular networks that regulate plant metabolism and stress-responsive pathways.^57^ ^,^ ^201^ Microbial-derived phytohormones and secondary metabolites influence antioxidant enzyme activities, osmolyte accumulation, nutrient transport, and ROS homeostasis while simultaneously activating systemic tolerance responses.^140^ ^,^ ^202^ ^,^ ^203^ In return, plants actively modify the composition of root exudates under abiotic stress, selectively recruiting beneficial microorganisms that reinforce these signaling networks and establish positive feedback mechanisms for stress adaptation.^204‐206^ This dynamic biochemical crosstalk enables plants to rapidly adjust their physiological functions and metabolic pathways, thereby improving the resilience and functional stability of the plant holobiont under changing environmental conditions.

### 6.3. Epigenetic regulation and gene expression

Microbial colonization reprograms plant gene expression and induces epigenetic modifications that regulate physiological adaptation to environmental stress.^207^ ^,^ ^208^ Beneficial microorganisms modulate the induction of stress-associated genes involved in antioxidant defense, ROS scavenging, ethylene signaling, osmotic adjustment, and photosynthesis, thereby improving plant tolerance under adverse environmental stresses.^109^ ^,^ ^193^ ^,^ ^194^ These transcriptional changes are coordinated through complex signaling pathways involving phytohormones, transcription factors, and chromatin remodeling, enabling plants to rapidly adjust their metabolism and cellular functions in response to abiotic stress.^209^ ^,^ ^210^ Representative examples include *Enterobacter* sp. SA-87 enhances thermotolerance through HSFA2-associated regulation of heat-responsive genes, whereas *B. subtilis* GB03, which regulates HKT1 expression under salt stress, and *Rhizophagus irregularis*, which improves nitrogen metabolism and drought tolerance through transcriptional regulation.^211‐213^

Emerging evidence further suggests that beneficial microorganisms contribute to stress tolerance by inducing stable epigenetic modifications, including DNA methylation, histone modifications, and chromatin remodeling, which influence the magnitude and persistence of plant stress responses.^214‐216^ These epigenetic changes enable plants to respond more rapidly and efficiently to recurring environmental stresses by maintaining a primed transcriptional state, thereby reducing the metabolic costs associated with repeated stress adaptation.^216^ ^,^ ^217^ Although the molecular basis of microbiome-induced epigenetic regulation remains incompletely understood, accumulating evidence indicates that this dynamic interaction between plant genomes and associated microorganisms is essential for sustaining the functional stability and adaptive capacity of the plant holobiont under changing environmental conditions.

### 6.4. From plant stress responses to holobiont resilience

Plant adaptation to abiotic stress is often associated with primed physiological, transcriptional, and epigenetic responses that enable more efficient responses to recurrent environmental stress.^218^ ^,^ ^219^ However, in natural and agricultural systems, these responses occur in close association with plant-associated microbiomes, which actively influence stress perception, biochemical signaling, gene expression, and microenvironmental conditions.^220^ ^,^ ^221^ Beneficial microorganisms may enhance plant stress adaptation by maintaining stress-adaptive microbial communities, producing signaling metabolites, modifying rhizosphere and phyllosphere microenvironments, and inducing long-lasting changes in host gene expression and epigenetic regulation.^222^ Therefore, stress adaptation can be viewed as an integrated holobiont-level phenomenon in which both the plant host and its symbiotic microbiota contribute to adaptive responses following previous stress exposure.^223^ Understanding this shift from a plant-centered perspective to a holobiont-based framework is key to advancing microbiome engineering strategies to improve crop resilience under recurrent abiotic stress.^224^ ^,^ ^225^

## 7. Microbiome engineering and synthetic microbial communities

### 7.1. SynCom design, functional complementarity, and plant benefits

The rational design and functional validation of SynComs are central to advancing agricultural microbiome applications.^226^ Developing a crop-specific, functionally optimized SynComs requires careful selection of strains that reflect the taxonomic composition and functional capacity of plant-associated microbiota, followed by rigorous performance evaluation under field conditions.^227‐229^ SynComs assembly strategies generally follow either bottom-up or top-down frameworks.^230^

In bottom-up approaches, individual strains are selected based on defined functional attributes and combined to generate emergent properties not achievable by single inoculants.^231^ Conversely, top-down strategies begin with complex native communities and reduce them to representative subsets that preserve key compositional and functional features.^232^ ^,^ ^233^ Empirical studies illustrate these principles, constructed a four-strain SynComs, comprising *Arthrobacter*, *Enterobacter*, *Brevibacterium*, and *Plantibacter* selected for complementary plant growth–promoting traits such as IAA production, phosphate solubilization, and antifungal activity against *Fusarium oxysporum.* ^234^ Greenhouse trials demonstrated that microbial consortium treatments promoted greater cotton growth than single-strain inoculations.^235^ Similarly, Yin et al.^236^ reported that SynComs assembled from wheat rhizosphere isolates suppressed *Rhizoctonia solani* more effectively than individual strains, highlighting the importance of synergistic microbial interactions.

Collectively, these findings demonstrate that rationally designed SynComs can provide multiple agronomic benefits beyond disease suppression, including enhanced nutrient acquisition, improved plant growth, and increased resilience under diverse environmental conditions.^231^ ^,^ ^237^ By integrating complementary functional traits from different microbial taxa, SynComs offer a more stable and effective alternative to single-strain inoculants, thereby supporting sustainable crop production while reducing reliance on synthetic agricultural inputs.^238‐240^ Representative examples of SynComs developed for different crop species, together with their functional traits and agricultural applications, are summarized in Table 2.

**Table 2.** Representative SynComs and microbiome engineering strategies relevant to plant resilience and holobiont adaptation.

Host crops	SynCom compositions	Main functional traits	Agricultural outcomes	References
Cotton	*Arthrobacter*, *Enterobacter*, *Brevibacterium*, *Plantibacter*	Plant growth promotion, phosphate solubilization, antifungal activity	Enhanced cotton growth compared with single-strain inoculants	Wang et al. [235]; Thomloudi et al. [241]
Wheat	Rhizosphere bacterial SynComs	Disease suppression	More effective suppression of *Rhizoctonia solani* than individual strains	Yin et al. [236]
Rice	*Variovorax paradoxus*, *Novosphingobium subterraneum*, *Hydrogenophaga pseudoflava*, *Acidovorax* sp.	Phosphorus acquisition and root development	Increased biomass, enhanced P uptake, improved expression of phosphate transporter genes	Ma et al. [242]
Maize	Sugarcane-derived bacterial SynComs	Drought tolerance	Reduced yield loss, improved water-use efficiency, lower leaf temperature, faster recovery after drought	Armanhi et al. [243]; Mondlhane et al. [244]

Crop-specific microbiome engineering is increasingly recognized as a promising strategy to translate microbiome functions into practical agricultural applications, including nutrient acquisition, stress resilience, and disease suppression in major crops.^245^ ^,^ ^246^ Compared with single-strain inoculants, synthetic microbial communities may provide broader and more stable functions because they combine complementary traits such as phytohormone production, phosphate solubilization, ACC deaminase activity, exopolysaccharide production, antioxidant regulation, and pathogen suppression.^247‐249^ These multifunctional traits are particularly relevant for strengthening holobiont adaptation because they may support the persistence of beneficial microbial functions and improve plant recovery after repeated environmental stress.^87^ ^,^ ^250^

### 7.2. Ecological and evolutionary constraints of SynCom establishment

The establishment of SynComs in field environments is constrained by environmental filtering, competition with resident microbiota, and priority effects, which can progressively reshape the introduced community.^251^ Soil properties, nutrient availability, host genotype, and environmental fluctuations shape microbial colonization and persistence, thereby determining which SynCom members can successfully establish under field conditions.^252^ Changes in these environmental conditions can alter the abundance and interactions of SynCom members, thereby reshaping community composition and the functional contributions of individual strains.^253^ These ecological processes can reduce the persistence and reproducibility of SynCom performance across heterogeneous field environments.^254^

Evolutionary processes can further modify SynCom composition and function over time.^255^ Intraspecific genetic variation and adaptation to local ecological conditions may alter traits associated with resource utilization, competition, host association, and microbial interactions, while horizontal gene transfer can contribute to changes in functional traits within microbial communities.^256^ ^,^ ^257^ Therefore, SynComs should be considered dynamic communities rather than fixed combinations of strains, with their long-term performance shaped by both ecological selection and evolutionary change.^255^ This creates a trade-off between maximizing specific plant-beneficial functions and maintaining ecological and evolutionary stability. Consequently, effective SynCom engineering should balance functional complementarity with strain adaptability, functional redundancy, and community stability, while considering how these properties change across environments and generations.

## 8. Future perspectives

Despite substantial advances in understanding plant–microbiome interactions under abiotic stress, several knowledge gaps remain before microbiome engineering can be widely translated into sustainable agricultural applications.^258^ ^,^ ^259^ A key research priority involves uncovering the molecular mechanisms that drive holobiont-level stress adaptation, through which previous stress exposure enables plants and their associated microbiota to respond more effectively to subsequent environmental challenges.^139^ ^,^ ^260^ Although microbe-mediated enhancement of plant stress resilience has been increasingly reported, the underlying mechanisms remain poorly understood.^261^ Future studies should investigate whether beneficial microbial colonization induces stable epigenetic modifications, including DNA methylation, histone remodeling, and chromatin reprogramming, that persist after stress exposure or are transmitted across generations.^262^ ^,^ ^263^ Integrating single-cell transcriptomics, epigenomics, and microbiome sequencing will provide deeper insights into how specific plant cell types perceive microbial signals and how these interactions contribute to long-term stress resilience.^264^ ^,^ ^265^

Advances in multi-omics technologies also provide new opportunities to accelerate microbiome engineering.^266^ Rather than relying on trial-and-error approaches, future SynComs should be designed using integrated metagenomic, metatranscriptomic, metaproteomic, and metabolomic datasets to identify microbial taxa, genes, and metabolites that consistently promote stress tolerance.^267‐269^ In parallel, artificial intelligence and machine learning models could predict optimal microbial consortia based on plant genotype, soil characteristics, climatic conditions, and microbial interaction networks.^176^ ^,^ ^270^ ^,^ ^271^ This transition from taxonomy-based to function-driven SynComs assembly, focusing on conserved stress-responsive traits such as ACC deaminase, exopolysaccharide biosynthesis, phytohormone production, and antioxidant metabolism, is expected to improve the robustness and field performance of engineered microbial communities.^272^ ^,^ ^273^

Microbiome engineering should move beyond generalized inoculant development toward crop-specific, compartment-specific, and function-driven strategies that account for plant genotype, soil properties, microbial interaction networks, and local environmental conditions.^224^ ^,^ ^274^ In this context, integrating metagenomics, metatranscriptomics, metabolomics, and artificial intelligence may help identify core microbial taxa and functional traits that consistently contribute to holobiont resilience, stress resilience, and field-level stability across diverse agroecosystems.^275‐277^

A major challenge for microbiome engineering remains its inconsistent performance under field conditions.^278^ Unlike controlled laboratory and greenhouse environments, agricultural fields are characterized by highly dynamic and heterogeneous conditions that strongly influence the establishment, persistence, and functionality of introduced microbial inoculants.^278^ Environmental factors such as fluctuations in temperature, soil moisture, pH, nutrient availability, and seasonal climate variability can significantly affect microbial survival and colonization efficiency.^278^ ^,^ ^279^ Moreover, intense competition with indigenous microbial communities, together with complex plant–soil–microbe interactions, often limits the ability of inoculated microorganisms to successfully establish and express their beneficial traits under natural conditions.^279^ ^,^ ^280^ These ecological constraints frequently result in variable biocontrol efficacy and inconsistent plant growth-promoting performance across different soils, climates, and cropping systems.^279^ ^,^ ^281^ Consequently, improving the field reliability of microbiome engineering requires strategies that account for microbial community dynamics, environmental adaptation, and host–microbiome interactions to achieve stable and sustainable agricultural outcomes.^280^ ^,^ ^281^

Therefore, future research should prioritize long-term multi-location field experiments to identify core stress-responsive microorganisms that function consistently across diverse agroecosystems.^282^ ^,^ ^283^ At the same time, improving microbial delivery systems, including seed coatings, bio-encapsulation technologies, and targeted rhizosphere formulations, will be essential for enhancing microbial survival and colonization.^284^ ^,^ ^285^ Beyond engineering microorganisms alone, increasing attention should also be directed toward developing microbiome-enabled crops through plant breeding, genome-wide association studies (GWAS), quantitative trait locus (QTL) mapping, and CRISPR/Cas-mediated genome editing to optimize root exudation, immune signaling, and microbiome recruitment under environmental stress.^286‐288^

Future research should further expand beyond the rhizosphere to include the phyllosphere and seed microbiomes, which also contribute to plant adaptation through the regulation of transpiration, photosynthetic performance, and early seedling establishment.^289^ ^,^ ^290^ Synthetic biology likewise offers exciting opportunities to develop programmable microbial biofactories capable of producing stress-protective compounds in response to specific environmental signals.^291^ However, these technological advances must be accompanied by comprehensive biosafety assessments, including evaluations of horizontal gene transfer, non-target ecological effects, and long-term impacts on soil microbial diversity, to support appropriate regulatory frameworks for field deployment.^292^ ^,^ ^293^

Finally, integrating microbiome engineering with digital agriculture technologies, including IoT-based environmental monitoring, remote sensing, portable sequencing platforms, and climate prediction models, may enable real-time monitoring of plant health and precision microbiome management.^271^ ^,^ ^291^ ^,^ ^294^ Collectively, these multidisciplinary approaches have the potential to transform microbiome engineering from descriptive ecological research into predictive, field-applicable technologies that support climate-resilient and sustainable agricultural production.

## 9. Conclusion

The plant microbiome is not simply a passive inhabitant but an active functional extension of the host that profoundly shapes stress resilience. This review synthesizes current evidence demonstrating that environmental challenges including drought, heat, and salinity trigger coordinated responses within the plant-microbe system, driving holobiont-level adaptation that can be harnessed through targeted engineering. Dysbiosis of these microbial networks exacerbates stress vulnerability, whereas strategic interventions such as SynComs, biofertilizers, and microbial biostimulants can restore ecological equilibrium and prime plants for enhanced tolerance. Mechanistically, beneficial microbes operate through multiple pathways: they produce osmoprotectants, scavenge ROS, modulate phytohormone signaling (e.g., ABA, ethylene), induce systemic tolerance, and even reprogram host gene expression via epigenetic modifications.

Notably, the “cry for help” strategy whereby stressed plants actively recruit stress-alleviating microbes, highlights the reciprocal, evolutionarily refined nature of these interactions. Collectively, microbiome-based approaches offer a sustainable, ecologically sound approach to conventional agrochemicals, with the potential to safeguard crop productivity under increasingly unpredictable climatic conditions. However, translating these promising laboratory and greenhouse findings into reliable field applications remains a central challenge, requiring deeper mechanistic understanding and robust technological platforms.

## Data Availability

All relevant data are included in the manuscript.
